# Automatic Detection of Dental Implant Brands and Exposure Status Using Deep Learning on Periapical Radiographs

**DOI:** 10.3390/diagnostics16213644

**Published:** 2026-11-04

**Authors:** Chih-Hao Chang, Yi-Cheng Mao, Chien-Yu Sung, Hsin-Kai Su, Kuo-Chen Li, Yuan-Jin Lin, Shih-Lun Chen, Da-Wei Hsu, Tsung-Yi Chen, Patricia Angela R. Abu, Wei-Chen Tu, Jia-Ching Wang

**Affiliations:** 1 Department of Computer Science and Information Engineering, National Central University, Taoyuan City 320317, Taiwan; 113582001@cc.ncu.edu.tw (C.-H.C.); jcw@csie.ncu.edu.tw (J.-C.W.); 2 Department of Operative Dentistry, Taoyuan Chang Gung Memorial Hospital, Taoyuan City 333008, Taiwan; 3 Department of Electronic Engineering, Chung Yuan Christian University, Taoyuan City 320314, Taiwan; s11226230@cycu.edu.tw (C.-Y.S.); s11226239@cycu.edu.tw (H.-K.S.); chrischen@cycu.edu.tw (S.-L.C.); g11402602@cycu.edu.tw (D.-W.H.); 4 Department of Information Management, Chung Yuan Christian University, Taoyuan City 320314, Taiwan; 5 Program on Semiconductor Manufacturing Technology, Academy of Innovative Semiconductor and Sustainable Manufacturing, National Cheng Kung University, Tainan City 701401, Taiwan; m28121562@gs.ncku.edu.tw (Y.-J.L.); wctu@gs.ncku.edu.tw (W.-C.T.); 6 Research Center for Semiconductor Materials and Advanced Optics, Chung Yuan Christian University, Taoyuan City 320314, Taiwan; 7 Department of Electronic Engineering, Feng Chia University, Taichung City 407102, Taiwan; tsungychen@fcu.edu.tw; 8 Ateneo Laboratory for Intelligent Visual Environments, Department of Information Systems and Computer Science, Ateneo de Manila University, Quezon City 1108, Philippines; pabu@ateneo.edu; 9 Department of Electrical Engineering, National Cheng Kung University, Tainan City 701401, Taiwan

**Keywords:** artificial intelligence, deep learning, implant detection, periapical radiograph

## Abstract

**Background/Objectives**: Dental implant technology has become the primary treatment modality for managing tooth loss in current clinical practice. Accurate identification of implant brands and their clinical exposure status have increasingly emerged as a critical issue in implant-related research and clinical diagnosis. Conventional approaches that rely on clinicians’ experience are time-consuming and may lead to misclassification due to variations in image quality or subtle differences in implant design features across brands. **Methods**: This single-center study classified six categories combining two implant brands (3i and Xive) and three exposure statuses. A retrospective cohort of 497 patients (708 periapical radiographs, 928 implant instances) was partitioned using an 8:1:1 stratified ratio with strict patient-level independence. Following YOLOv8-based implant localization and ROI extraction, classification was evaluated across deep learning backbones using 5-fold cross-validation and compared against a panel of three dental specialists on a test set of 8 complex cases. **Results**: YOLOv8 achieved a precision of 99.39% and a sensitivity of 98.63% in the implant detection task. For the classification of implant brand and exposure status, EfficientNet-B3 achieved the best performance with an accuracy of 99.18 ± 0.85% (95% CI: [94.20%, 100.00%]) across 5-fold cross-validation, demonstrating competitive diagnostic performance alongside existing implant classification benchmarks in the literature. In a preliminary clinical feasibility assessment on representative cases, the framework demonstrated rapid algorithmic inference (mean inference time of 36.18 ms per image), providing instant decision-support potential alongside participating clinicians. **Conclusions**: This study demonstrates the technical feasibility and proof-of-concept of a deep learning framework for identifying dental implant brands and exposure status under robust internal validation. While offering preliminary decision-support potential, external validation on multi-center cohorts and prospective clinical trials across expanded implant systems remain necessary before clinical implementation.

## 1. Introduction

With advances in dental treatment technologies, dental implants have become the global gold standard for tooth replacement [1]. With global population aging and increasing awareness of oral health, the number of implants placed continues to rise steadily, making them an indispensable treatment modality in contemporary dental rehabilitation [2]. However, as manufacturers continuously introduce new designs, the number of implant brands and specifications available on the market has now exceeded several hundred, with significant variations arising from differences in thread morphology, neck structure, and connection systems [3]. In clinical follow-up, restorative procedures, or complication management, the absence of complete medical records poses considerable challenges for clinicians in identifying implant brands and structural features [4]. Beyond brand recognition, the determination of exposure status is also of critical clinical importance, as it is closely associated with implant long-term stability, the risk of peri-implant bone resorption, and subsequent restorative or complication management [5,6]. Specifically, identifying whether an implant is at the submerged stage (requiring surgical uncovering), the healing cap stage (ready for soft-tissue contouring and restorative impressions), or the definitive prosthesis stage (requiring maintenance and complication management) provides actionable guidance for chairside clinical workflows. Furthermore, from a computer vision perspective, overlying healing abutments and restorative crowns alter the coronal profile on radiographs; concurrently classifying the exposure status prevents the deep learning model from confusing prosthetic components with intrinsic brand-specific designs. The integration of implant brand identification with exposure status determination could provide more comprehensive and clinically meaningful decision-support information, improving diagnostic accuracy and efficiency.

The rapid advancement of artificial intelligence (AI) and deep learning algorithms has driven significant progress in computer-aided diagnosis within dentistry. Current AI models have demonstrated high efficacy in tasks such as root canal [7], oral mucosal lesion [8], inferior alveolar nerve [9], peri-implantitis [10,11], tooth wear severity [12], tooth-numbering [13], and marginal ridge discrepancy [14], which are among the key clinical concerns in dentistry [15,16]. However, studies that simultaneously apply deep learning to identify implant brands and assess their clinical status remain scarce. Most of the existing literature focuses solely on binary or multi-class brand classification. For instance, while Lin et al. [17] successfully employed an object detection model to identify implant brands on periapical radiographs (PAs) with 94.5% accuracy, their study did not evaluate the implant’s exposure conditions. Similarly, Dashti et al. [18] demonstrated that deep learning approaches can effectively identify implant brands to support treatment planning. In addition, Zitzmann et al. [19] and Dos Santos et al. [20] highlighted that distinguishing implant conditions—such as emerged fixtures, healing caps, and prostheses—is essential for clinical evaluation, stress analysis, and long-term postoperative maintenance. Here, “exposure status” (or clinical restorative stage) specifically refers to the presence and type of transgingival or prosthetic components attached to the implant platform visible on radiographs: (1) emerged (fixture-only or cover screw stage), where the implant is osseointegrated in bone without transgingival components; (2) healing cap, where a transmucosal healing abutment is connected; and (3) prosthesis, where the definitive abutment and restoration are fully installed. Moreover, an integrated automated framework capable of delivering these concurrent assessments has not been actualized. Relying solely on brand classification limits the clinical utility of these AI models, as clinicians require a comprehensive radiographic evaluation—specifically, the simultaneous assessment of both the implant fixture brand and its connected coronal restorative component (treatment stage)—to determine appropriate and immediate clinical interventions without ambiguity.

To address this crucial clinical gap, this study proposes an automated AI-assisted diagnostic framework that integrates object detection with image classification networks. The primary research question of this study is: Can a two-step deep learning framework achieve high-accuracy classification of six distinct dental implant categories, comprehensively integrating various brand identities with their corresponding exposure statuses, using standard clinical periapical radiographs? This study hypothesized that this unified six-class integrated model would demonstrate robust diagnostic performance, comparable to or exceeding that of clinical practitioners. Therefore, the specific objectives of this study were to: (1) develop and train a two-step AI model capable of accurately classifying these six specific implant categories on PAs; and (2) evaluate the model’s generalizability, accuracy, and robustness by comparing its diagnostic outcomes against the evaluations of experienced clinical dentists.

## 2. Materials and Methods

**Figure 1:**
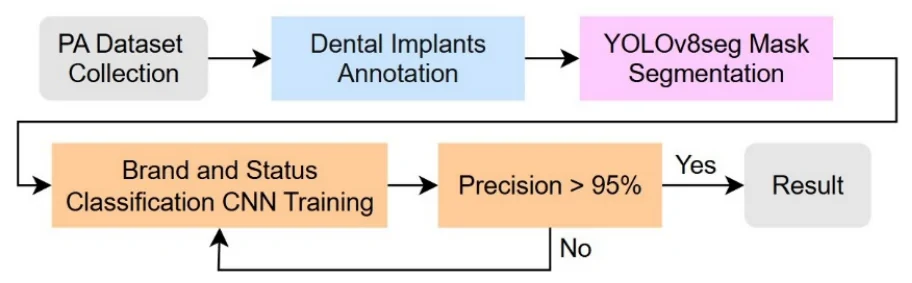
Main flow chart for AI-assisted diagnostic framework.

This section introduces the experimental procedures and methodologies adopted in this study, with the detailed workflow illustrated in Figure 1**.** First, dental experts annotated the ground truth for implant brands and exposure status. The proposed deep learning architecture operates through a four-stage sequential pipeline: (1) Localization and Instance Segmentation, where YOLOv8s-seg identifies the implant complex; (2) Pixel-level Mask Generation, delineating the implant boundary against surrounding bone trabeculae; (3) ROI Extraction, cropping the isolated implant region based on tight bounding coordinates; and (4) Two-Task Classification, where standardized ROIs (224 × 224 × 3) are fed into downstream Convolutional Neural Network (CNN) backbones (e.g., ConvNeXt_v2) to concurrently classify brand identity and clinical exposure status across six composite categories.

### 2.1. Implant Brand Dataset Collection

**Figure 2:**
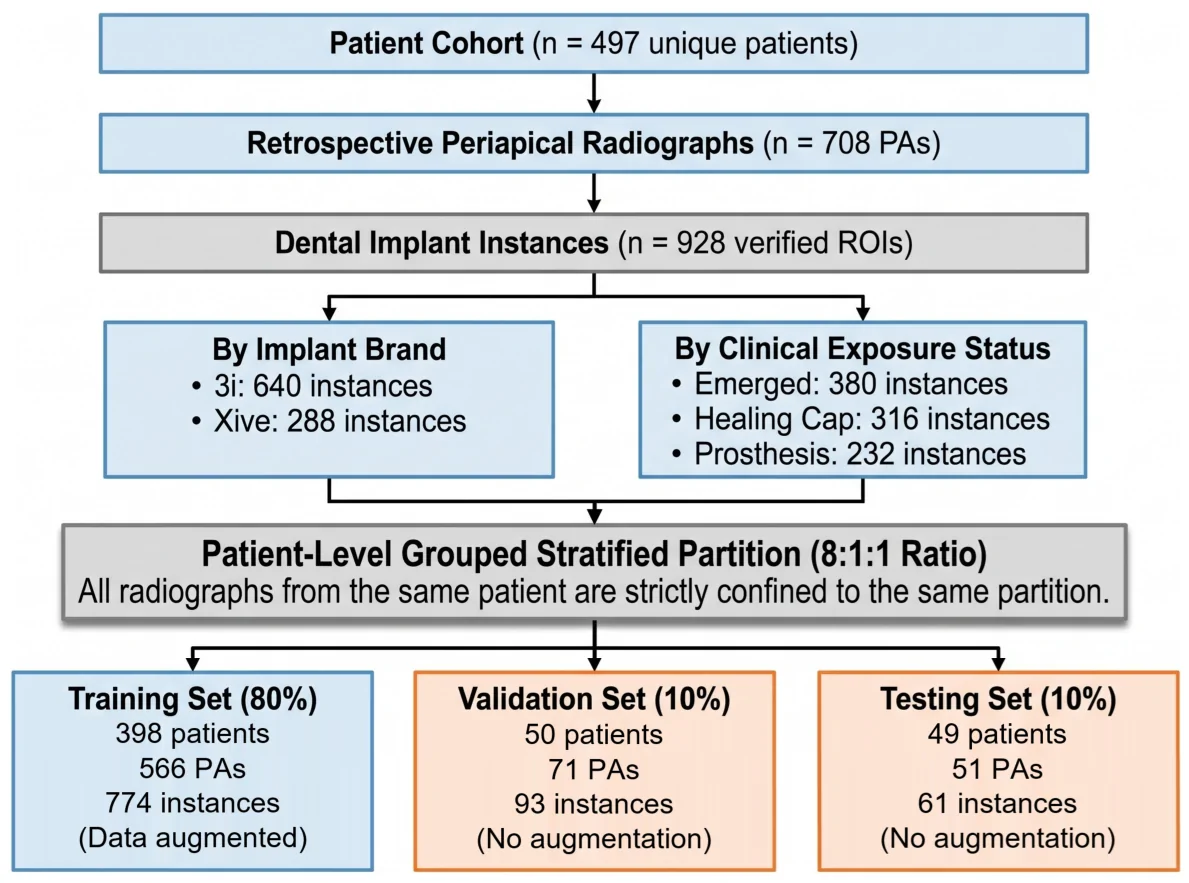
Study Flow Diagram and Dataset Partitioning.

The PA dataset was provided by Chang Gung Memorial Hospital in Taoyuan, Taiwan, and approved by the Institutional Review Board (IRB) of the Chang Gung Medical Foundation (IRB number: 202500009B0), with a waiver of informed consent. Data collection and annotation were performed by three dental specialists, each possessing over five years of clinical implantology experience. To establish an unequivocal reference ground truth for implant brands, identities (3i and Xive) were definitively verified from institutional electronic medical records, operative surgical logs, and manufacturer product barcodes rather than visual guesswork. The exposure status was independently determined by the three blinded specialists according to predefined radiographic criteria: (1) emerged (fixture-only or cover screw without supragingival projection), (2) healing cap (cylindrical transmucosal abutment), and (3) prosthesis (definitive abutment and crown restoration). Any diagnostic discrepancies were resolved through mutual consensus supervised by a senior prosthodontic specialist. Periapical radiographs were retrospectively retrieved from the institutional dental Picture Archiving and Communication System (PACS) covering examinations conducted between 2018 and 2023. All images were acquired during routine clinical practice using institutional high-frequency intraoral X-ray units and digital radiography sensors under standard diagnostic exposure settings (60–70 kVp, 6–8 mA). The raw digital radiographs were exported in standardized 8-bit uncompressed format, with image matrix sizes ranging from 1200 × 1600 to 1440 × 1920 pixels (effective spatial resolution ~18 lp/mm). The included patients ranged in age from 20 to 65 years, and each PA contained at least one dental implant. Radiographs were excluded if they exhibited: (1) severe geometric distortion or extreme projection angulation that obscured the implant thread geometry, (2) prominent motion blur or unreadable contrast artifacts, or (3) incomplete visualization of the implant fixture and its cervical platform. The dataset was collected from 497 patients and consisted of 708 PAs, comprising 640 implant instances with 3i implants and 288 instances with Xive implants (totaling 928 implant instances). Regarding implant exposure status, the implants were categorized into three distinct clinical stages according to the radiographic components attached to the implant platform: (1) emerged (or fixture-only/cover screw stage; 380 instances), showing only the implant fixture or a flat cover screw without transmucosal components; (2) healing cap stage (316 instances), displaying a transmucosal healing abutment; and (3) prosthesis stage (232 instances), exhibiting the definitive abutment and crown restoration. The overall recruitment workflow, hierarchical sample accounting, and dataset allocation across subsets are summarized in Figure 2.

### 2.2. YOLOv8 Segmentation

**Figure 3:**
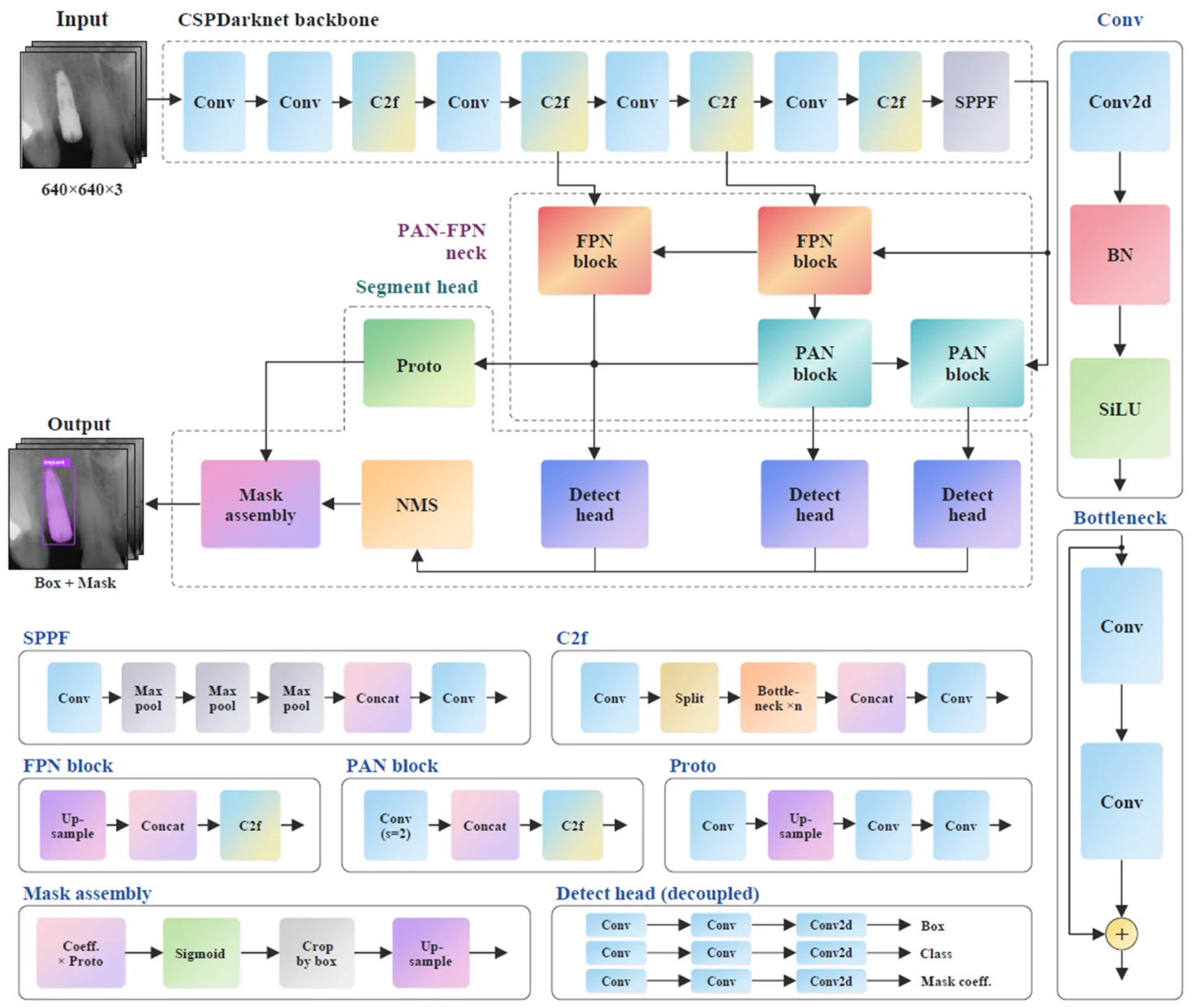
Architectural pipeline of YOLOv8s-seg for implant localization and instance segmentation.

In this framework, YOLOv8s-seg (Ultralytics Inc., Frederick, MD, USA; https://www.ultralytics.com/) was employed strictly for implant localization and instance segmentation to extract regions of interest (ROIs), rather than for final classification. Architecturally, it utilizes the C2f (Cross-Stage Partial Bottleneck with 2 Convolutions) module in its backbone to optimize multi-scale feature extraction for accurate boundary delineation against alveolar bone. In the annotation protocol, bounding regions covered the complete implant-restorative complex, encompassing the implant fixture, transmucosal components (healing caps or abutments), and prosthetic crowns. This inclusion was necessary because our downstream CNN performs concurrent classification of both implant brand and exposure status; excluding coronal superstructures would eliminate the visual features required to distinguish restorative stages (emerged vs. healing cap vs. prosthesis). Furthermore, preserving the coronal assembly maintains the natural fixture–abutment connection silhouette, which provides auxiliary architectural cues without artificially cropping critical cervical brand features. Data annotation was performed using the online platform Roboflow (Roboflow Inc., Des Moines, IA, USA; https://roboflow.com). The dataset was partitioned using an 8:1:1 stratified ratio into training (398 unique patients, 566 PAs, 774 implant instances), validation (50 unique patients, 71 PAs, 93 implant instances), and test sets (49 unique patients, 51 PAs, 61 implant instances). To confirm data partition integrity and prevent patient-level data leakage, a comprehensive patient-level audit was conducted. All radiographs from any given patient were strictly confined to a single partition, with zero patient overlap across the training, validation, and testing subsets. Data augmentation strategies (including horizontal flipping, slight rotations within ±10^∘^, and brightness adjustments) were applied exclusively to the training set after data partitioning, while the validation and test sets remained completely unaugmented. In addition, validation loss was monitored during training, and the model weights corresponding to the best validation performance were preserved to prevent model overfitting. During training, the input size was set to 640 × 640 pixels, the number of epochs was set to 100, the batch size was 4, and the initial learning rate was 0.0001. The detailed network architecture, incorporating CSPDarknet backbone, C2f modules, PAN-FPN neck, and the decoupled segment head, is illustrated in Figure 3. While YOLOv8s-seg served as the primary localization and segmentation engine, recent state-of-the-art variants (YOLOv10, YOLOv11, and YOLOv12) were concurrently implemented under identical training hyperparameters and dataset splits to establish a comparative benchmark in Section 3.1.

### 2.3. Convolution Neural Network Classification

**Figure 4:**
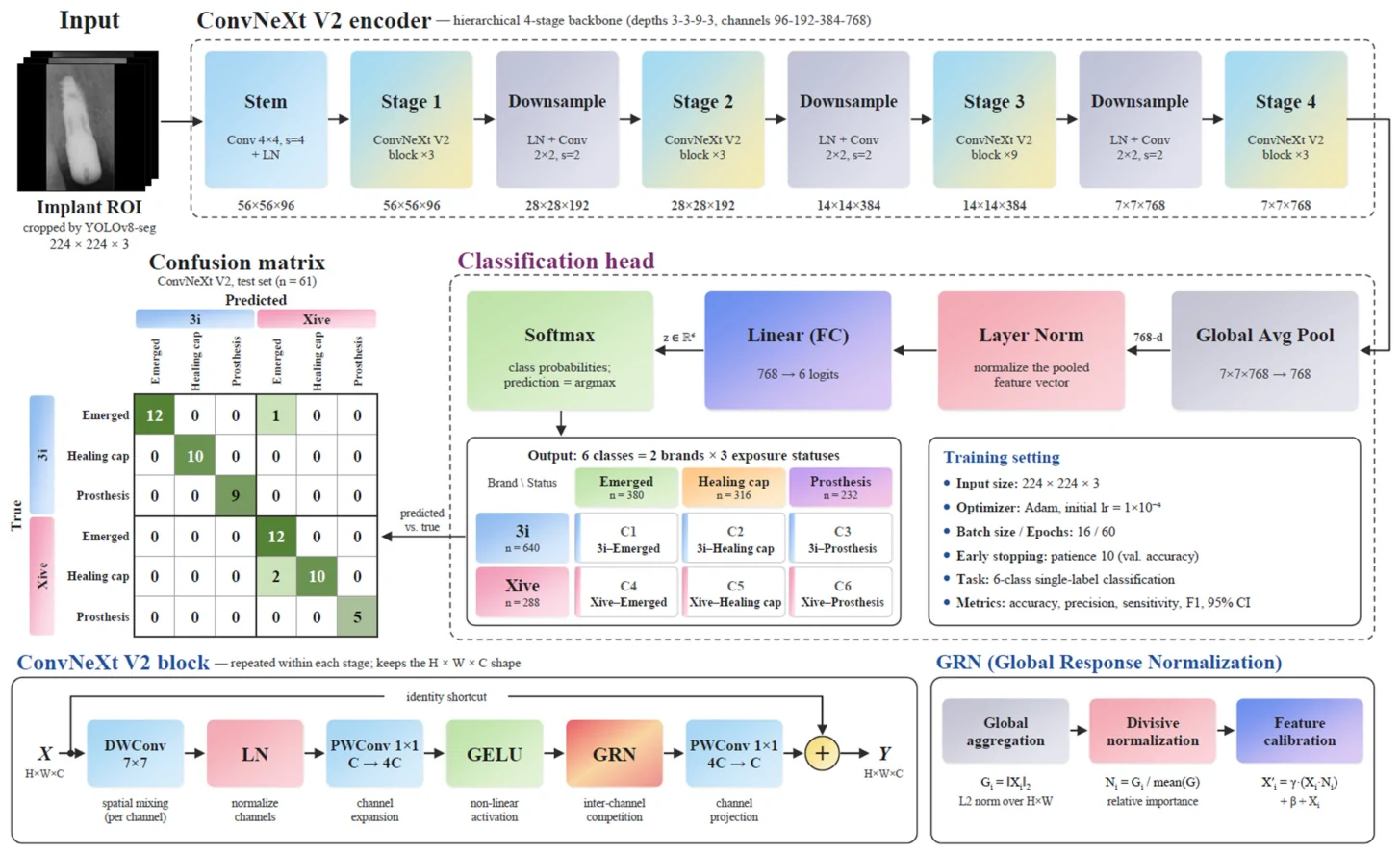
Detailed architectural overview of the ConvNeXt V2 classification framework.

Multiple CNN architectures were employed to perform two classification tasks simultaneously: identifying (1) implant brand identity (3i vs. Xive) and (2) clinical exposure status (emerged, healing cap, or prosthesis), which were integrated into six combined categories (3i-emerged, 3i-healing cap, 3i-prosthesis, Xive-emerged, Xive-healing cap, and Xive-prosthesis). The models used included ResNet50, EfficientNet-B3, MobileNet_v3, DenseNet121, and ConvNeXt_v2. These networks can effectively classify complex imaging data by leveraging deep feature extraction and efficient network designs. Unlike conventional machine learning methods, CNN models can automatically learn spatial representations from images and have demonstrated superior performance in implant brand and exposure status classification tasks. An early stopping strategy was applied to prevent overfitting, whereby training was terminated if the validation accuracy did not improve over 10 consecutive epochs. The detailed architectural pipeline, block-level design of ConvNeXt V2, training configurations, and the resulting classification mapping are illustrated in Figure 4. Specifically, cropped implant regions of interest (ROIs, 224 × 224 × 3) obtained via YOLOv8s-seg are fed into a 4-stage hierarchical backbone incorporating ConvNeXt V2 blocks with Global Response Normalization (GRN). The classification head subsequently pools feature representations into a 6-class output space covering two clinical attributes (2 brands × 3 exposure statuses). To ensure a fair and rigorous comparative evaluation, all candidate classification architectures were trained under strictly identical experimental conditions and hyperparameter regimes. In this phase, the input size was set to 224 × 224 pixels, and the Adam optimizer was employed for model weight updates. During preliminary model optimization, an iterative hyperparameter tuning loop was evaluated strictly on the validation set using a precision threshold of 95% as an operational gate (Figure 1). Across 64 exploratory trials systematically tuning the initial learning rate (1 × 10^−3^ to 1 × 10^−4^), batch size (8 to 32), and epochs (30 to 60), the optimal training configuration meeting this criterion was established (initial learning rate = 0.0001, batch size = 16, epochs = 60). Final model training was subsequently executed with an early stopping strategy (monitoring validation accuracy with a patience of 10 epochs) to prevent overfitting, strictly isolating the independent test set from the tuning process.

All classification backbones were initialized using pre-trained weights before fine-tuning. Model training, validation, and inference latency benchmarks were executed on a dedicated workstation equipped with an Intel Core Ultra 7 265K CPU (Intel Corporation, Santa Clara, CA, USA), 32 GB RAM, and an NVIDIA GeForce RTX 4080 GPU (16 GB VRA; NVIDIA Corporation, Santa Clara, CA, USAM) running 64-bit Windows 11 (Microsoft Corporation, Redmond, WA, USA), PyTorch 2.2.0, and CUDA 12.1.

### 2.4. Evaluation Metrics

We used different metrics ((1)–(6)) to evaluate YOLO and CNN reliability and stability. The metrics include Accuracy, Precision, Recall (Sensitivity), mean Average Precision (mAP), F1-score and used receiver operating characteristics curve (ROC) to evaluate the confidence intervals (95% CI). TP (true positive), FP (false positive), TN (true negative) and FN (false negative) are defined by confusion matrix [21]. For multi-class classification across the six categories, overall Precision, Sensitivity, and F1-score were aggregated using macro-averaging. Specifically, for evaluating ROC curves and AUC values in the YOLO object detection setting, predictions were matched to reference ground truth using an Intersection over Union (IoU) threshold of 0.50. Detections with IoU ≥ 0.50 were designated as TP, whereas predictions with IoU < 0.50 or redundant detections were treated as FP, with true positive rates (TPR) and false positive rates (FPR) computed across varying confidence thresholds from 0 to 1 via numerical integration. Moreover, $\tilde{x}$ represented sample mean, s was the sample standard deviation, n was the sample size, and t_a/2,df_ was the t-score corresponding to the desired confidence level and degrees of freedom for 95% CI.

(1) $$\mathrm{Accuracy}=\frac{\mathrm{TP}+\mathrm{TN}}{\mathrm{TP}+\mathrm{TN}+\mathrm{FP}+\mathrm{FN}}$$

(2) $$\mathrm{Precision}=\frac{\mathrm{TP}}{\mathrm{TP}+\mathrm{FP}}$$

(3) $$\mathrm{Recall}(\mathrm{Sensitivity})=\frac{\mathrm{TP}}{\mathrm{TP}+\mathrm{FN}}$$

(4) mAP=1n∑i=1n APi

(5) $$F1-\mathrm{score}=\frac{2\times \mathrm{Precision}\times \mathrm{Recall}}{\mathrm{Precision}+\mathrm{Recall}}$$

(6) $$95\%\mathrm{CI}=\tilde{x}\pm t_{a/2,\mathrm{df}}\times \frac{s}{\sqrt{n}}$$

To address the class imbalance between Biomet 3i and Dentsply Xive samples during training, an inverse frequency-weighted cross-entropy loss was employed alongside targeted data augmentation, and all evaluation metrics were computed independently for each class to assess balanced diagnostic performance.

## 3. Results

### 3.1. Implant Object Detection Experiment and Results

**Figure 5:**
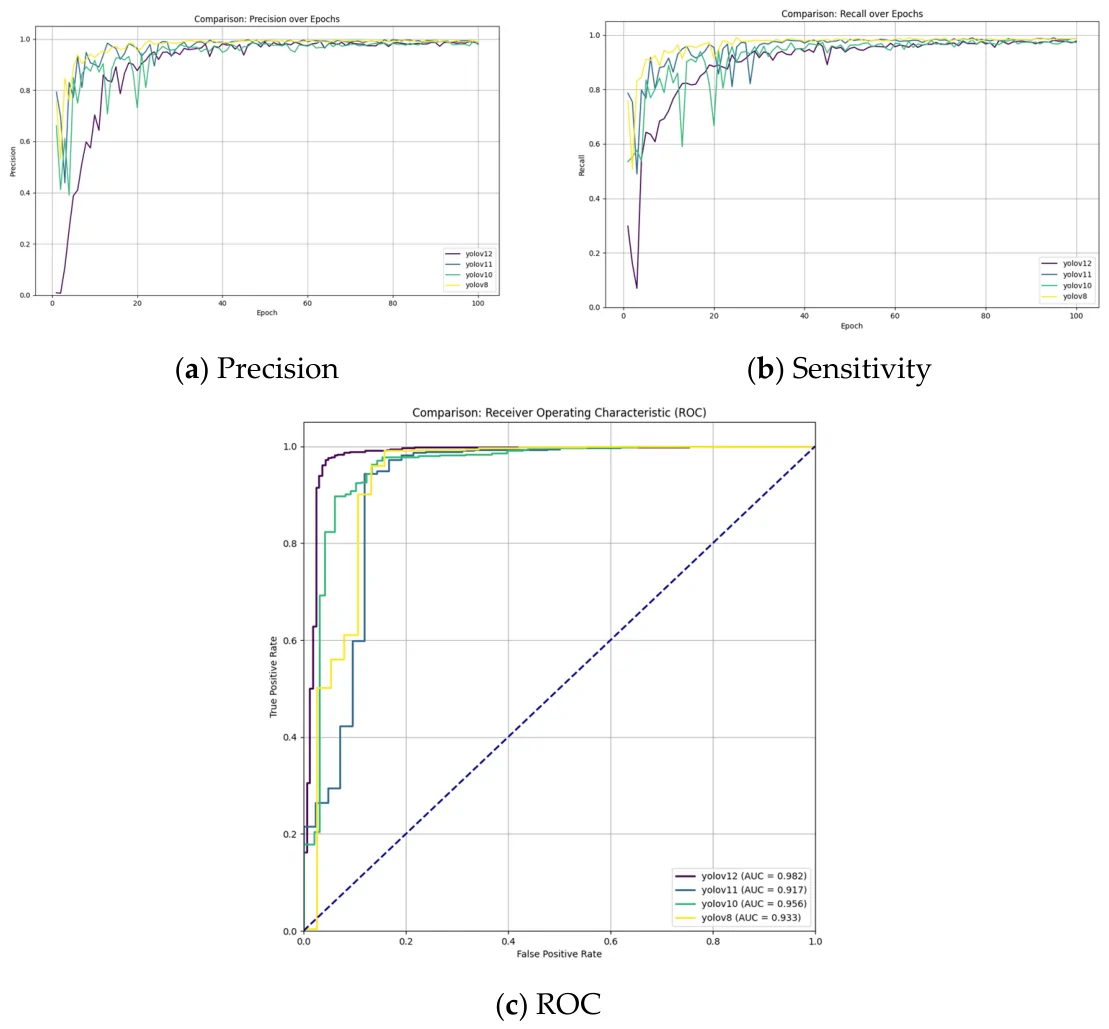
Training and validation progression of candidate YOLO models for implant localization and detection (evaluated on the training set [*n* = 774 instances] and validation set [*n* = 93 instances]).

This subsection evaluates and compares the performance of multiple YOLO object detection models for automated dental implant recognition. Figure 5 illustrates the training processes of the four models. All models exhibited rapid convergence, stabilizing performance metrics after approximately 40 epochs. Among them, YOLOv8 demonstrated the fastest and smoothest rise in its learning curve during the early training phase, whereas YOLOv12 showed greater fluctuations at the beginning but eventually converged to a performance level comparable to the other models. In addition, we compared ROC curves of the four models and calculated the Area Under the Curve (AUC) values. The results showed that YOLOv12 achieved the highest AUC value of 98.2%, indicating the strongest ability to distinguish true positives from false positives across all confidence thresholds. This was followed by YOLOv10 (AUC 95.6%), while YOLOv11 and YOLOv8 achieved AUC values of 91.7% and 93.3%. Although YOLOv12 achieved a marginally higher AUC on the validation cohort, YOLOv8 was adopted as the foundational upstream detector due to its superior overall precision (99.39%), robust localization under stringent IoU thresholds (mAP_50-95_ = 80.05%, Table 1), and proven edge-deployment stability within our sequential real-time pipeline.

Table 1 summarizes the best performance metrics achieved by each model on the test set. All models achieved an mAP50 above 98.9%, demonstrating highly stable detection capability. Among them, YOLOv8 achieved the best performance in both precision and sensitivity, reaching 99.39% and 98.63%, respectively, indicating a well-balanced ability to minimize false positives while maintaining a high detection rate. In contrast, YOLOv10 achieved a precision of 99.13%, but its sensitivity was only 96.71%, reflecting high precision (though slightly lower than YOLOv8’s 99.39%) but a greater tendency to miss certain implants. YOLOv11 achieved the best result on the mAP50-95 metric (81.10%), with precision and sensitivity of 99.12% and 98.62%, respectively, suggesting superior generalization ability across varying IoU thresholds. YOLOv12 showed relatively lower overall performance, with an mAP50-95 of 77.48%, precision of 99.02%, and sensitivity of 97.50%, maintaining high accuracy but with a slightly reduced detection rate compared with YOLOv8 and YOLOv11. YOLOv8 demonstrated the most balanced and superior performance regarding precision and sensitivity, while YOLOv11 showed advantages in generalization ability. YOLOv10 and YOLOv12 maintained high precision but were comparatively weaker in sensitivity or generalization. We conducted a five-fold cross-validation to confirm model robustness, and YOLOv8 achieved the highest 95% CI of [96.72%, 99.92%]. Notably, because different YOLO architectures employ distinct mathematical loss formulations, reported training loss values demonstrate intra-model optimization convergence rather than direct cross-architecture quantitative equivalence.

### 3.2. Implant Brand and Status Classification Experiment and Result

**Figure 6:**
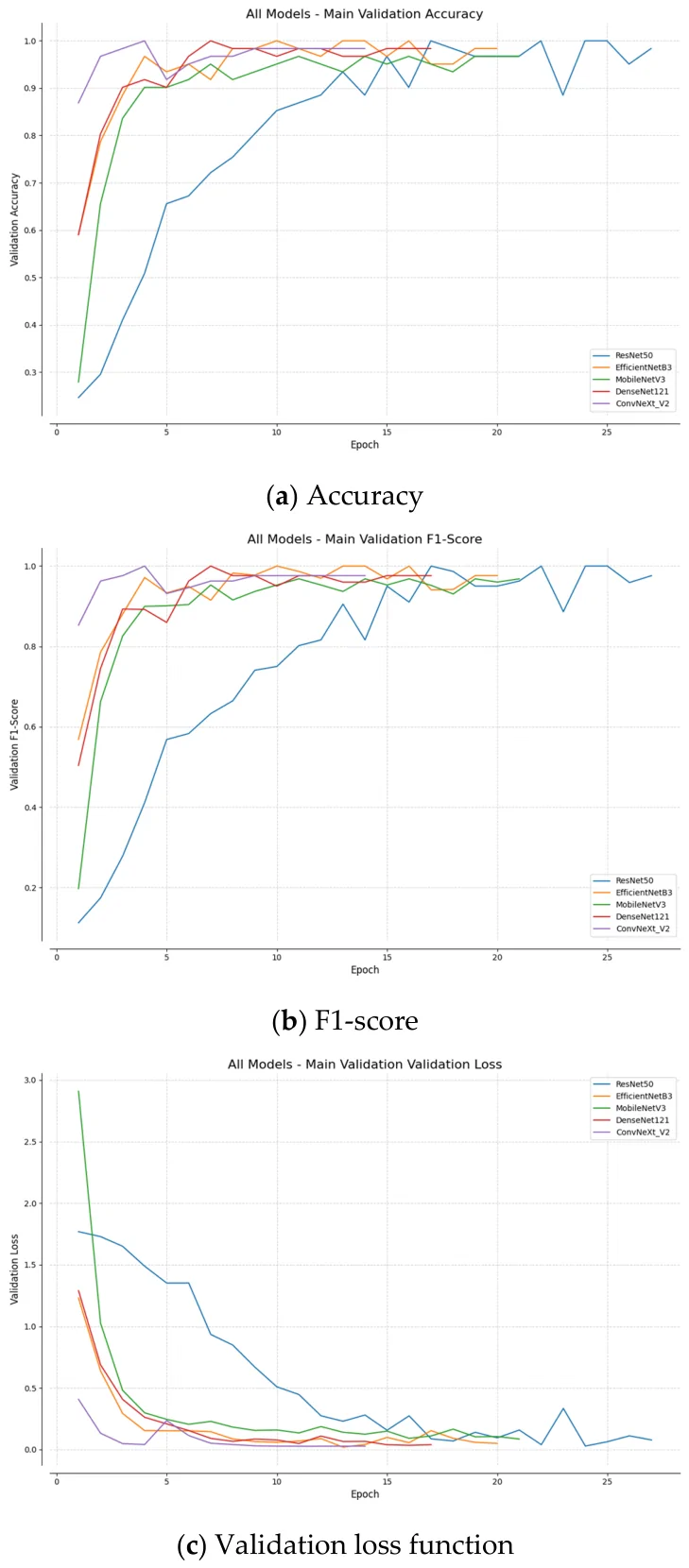
CNN training processing for implant brand and status classification.

This subsection evaluates the performance of five models on the two classification tasks, with the results summarized in Table 2. The findings show that EfficientNet-B3 achieved the most outstanding and robust performance, with an accuracy of 99.18 ± 0.85%, precision of 99.25 ± 0.76%, sensitivity of 99.10 ± 0.92%, and F1-score of 99.17 ± 0.81% across 5-fold cross-validation (95% CI for accuracy: [94.20%, 100.00%]). MobileNet_v3 and ResNet50 also achieved strong results, with all metrics consistently above 98.8%. Although ConvNeXt_v2 exhibited a slightly lower sensitivity (97.22 ± 1.20%), its F1-score remained high at 97.77 ± 1.12%. Figure 6 illustrates the changes in validation loss, accuracy, and F1-score during the training process for all five models. The results demonstrate that all models exhibited rapid convergence. ConvNeXt_v2 converged the fastest, reaching stable performance within approximately five epochs. In contrast, ResNet50 showed a smoother but less stable learning curve, with notable fluctuations during the later training stages. EfficientNet-B3, MobileNet_v3, and DenseNet121 demonstrated stable and efficient learning trends throughout training. Evaluating performance per class confirmed that the framework maintained balanced accuracy despite sample size disparities: Biomet 3i yielded a precision of 99.41 ± 0.62%, recall of 99.35 ± 0.71%, and F1-score of 99.38 ± 0.54%, whereas Dentsply Xive achieved a precision of 98.72 ± 1.15%, recall of 98.84 ± 1.08%, and F1-score of 98.78 ± 0.92%.

**Figure 7:**
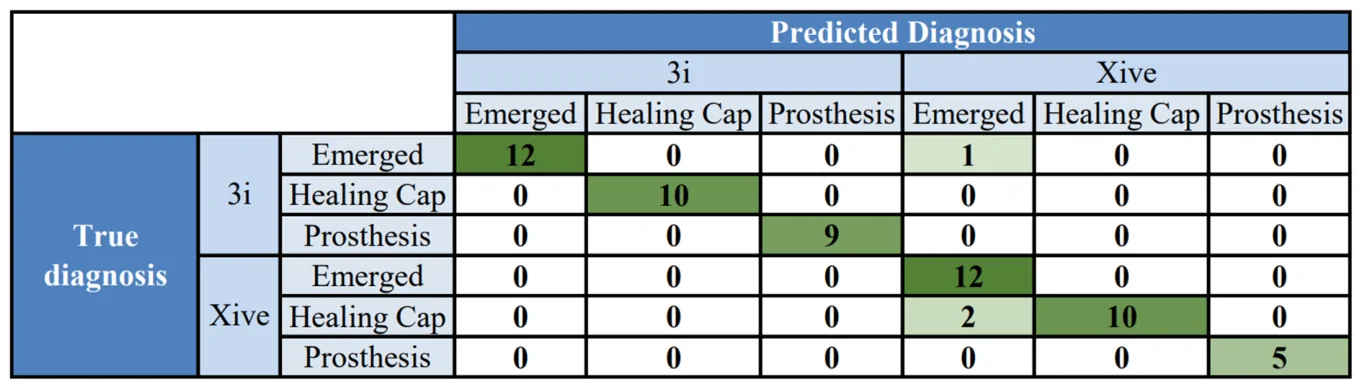
ConvNeXt_v2 confusion matrix on the independent test set (*n* = 61).

Figure 7 presents the confusion matrix of ConvNeXt_v2 on the test set. The diagonal elements represent the number of correctly classified samples, with 58 correctly classified and 3 misclassified instances (test accuracy: 95.08%). The main classification errors were observed in a small number of cases where 3i samples were misclassified as Xive (one instance of 3i-Emerged predicted as Xive-Emerged), and within the Xive brand, where two instances of Healing Cap were misclassified as Emerged. Despite these isolated misclassifications, the confusion matrix demonstrates that the model achieved exceptionally high accuracy in the complex classification task involving six combined categories.

### 3.3. Clinical Ground Truth Comparison

This subsection evaluates the clinical feasibility of our AI-assisted framework in comparison with three dental specialists, each having over five years of clinical experience. The evaluation was conducted independently and blindly under standardized viewing conditions, recording the predicted brand and status, reading time, and diagnostic confidence. Inter-rater reliability among the three evaluators showed excellent agreement, with an intraclass correlation coefficient (ICC) of 0.938. The comparative results between ground truth, manual annotation, and AI inference are summarized in Table 3. All the implant brand and exposure status can be judged collectively, and our proposed AI-assisted framework achieved a mean confidence score of 99.72% with a mean inference time of 36.18 ms. In comparison, the three dentists required an average of 8.79 s per radiograph. The 243-fold reduction in evaluation time was derived from the relative speed improvement ratio, calculated as (*T*_dentist_ − *T*_AI_)/*T*_AI_ ≈ (8.79 s − 0.03618 s)/0.03618 s ≈ 242.06, highlighting the real-time inference capability of the framework. We explicitly note that raw algorithmic inference latency and human clinical reading duration characterize distinct operational processes, and this numerical ratio serves strictly to highlight computational efficiency rather than an absolute reduction in comprehensive chairside consultation time.

## 4. Discussion

Implant brand and exposure status play a critical role in clinical treatment, particularly when medical records are unavailable or patients are referred from other institutions. Accurate identification of implant type is essential for subsequent restorative procedures and complication management. Failure to clearly recognize the three common implant exposure statuses may compromise clinicians’ ability to determine the appropriate treatment stage and intervention strategy. Traditionally, the identification of implant brand and status has relied heavily on clinicians’ experience and visual assessment. However, variations in image quality, inconsistencies in radiographic angles, and subtle similarities in implant design often led to interpretation errors, reducing diagnostic accuracy and consistency. With the increasing diversity of implant types and design specifications, distinguishing between different brands and classifying their exposure status has become increasingly challenging and time-consuming. This study developed an AI system based on deep learning that automatically identifies implant brands and classifies their exposure status from radiographic images. This system provides clinicians with accurate and consistent decision-support information, and it can also be applied to routine monitoring for long-term implant follow-up and complication management.

Most existing studies have adopted deep learning models for semi-automated detection of implant brands and exposure status. Considering the difficulty in collecting sufficiently large datasets for training, most of these works employed CNN-based architectures that are more suitable for small-scale datasets, achieving high accuracy. For example, Guo et al. [22] utilized 600 PAs with a modified VGG16 network to identify three commonly used implant brands in China, achieving an accuracy of 78.7% and a precision of 82%. Jang et al. trained Faster R-CNN on 300 PAs for implant brand detection in Korea [23], reporting a precision of 97.7%, while noting potential misclassification caused by small datasets and blurred implant boundaries. Saïd et al. applied GoogleNet to classify six commonly used implant brands in France [24], achieving an accuracy of 93.8%. Kohlakala et al. employed a fully convolutional network to classify 82 images containing four implant brands from South Africa and Belgium, reaching an accuracy of 90.43% [25]. Sukegawa et al. [26] classified 13 implant brands from 1091 pre-segmented implant images in Japan, highlighting the limitation of semi-automated approaches where implant regions were not extracted directly from dental radiographs before brand identification.

In contrast, the present study utilized a fully automated framework based on PAs collected from Chang Gung Memorial Hospital in Taoyuan, Taiwan, to classify implant brands and exposure status. Compared with the two-class classification of 3i and Xive implants by Lin et al. [17], which achieved 94.5% accuracy, our approach, integrating YOLOv8 with EfficientNet-B3, achieved a robust cross-validated accuracy of 99.18 ± 0.85% across six combined categories, demonstrating competitive diagnostic performance in a descriptive cross-study comparison. However, direct comparison across different studies requires an appropriate disclaimer: variations in sample composition, class granularity (e.g., binary brand classification versus our 6-class composite scheme), and evaluation protocols inherently limit strict numerical equivalence. Recent multicenter investigations have advanced toward multi-class evaluations across broader implant libraries; for example, Lee et al. [27] and Kim et al. [28] evaluated deep learning models across numerous commercial implant systems on multi-institutional datasets, demonstrating that larger class numbers capture clinical diversity but face challenges from inter-center radiographic variations and data imbalance. Contextualized within this landscape, our framework validates the technical feasibility of simultaneous brand and exposure status determination as a foundational proof-of-concept. Regarding exposure status classification, Lashaki et al. combined VGG16 features with traditional classifiers in a three-class task [29], achieving 89.66% accuracy and 93.64% precision, demonstrating the stability of traditional models on small datasets. Park et al. reported that classification accuracy remained at 88% even when three status categories were included without explicit labeling [30], suggesting that models can still distinguish among the three stages based on external appearances. Similarly, Sukegawa et al. categorized implant status into three groups while simultaneously identifying 12 implant brands using CNNs [31], achieving an F1-score of 93% in 4-fold cross-validation. Their findings further indicated that larger parameter counts, and deeper networks generally yield better brand and status classification performance. In comparison, as demonstrated by the multi-class evaluation in Section 3.2, our unified framework achieves superior accuracy and robustness across both exposure status and brand identification, highlighting its potential clinical applicability in assisting dentists with implant evaluation.

Despite developing a high-performance automated system for dental implant analysis in this study, several limitations remain to be addressed. Acknowledging these shortcomings provides a more comprehensive understanding of the study’s conclusions and highlights key directions for future research. First, the single-source nature of the dataset represents an important limitation, as the implant cases were collected from a single institutional repository restricted to the Taiwanese population without external multi-center validation. Moreover, although hundreds of implant systems exist worldwide, this study focused on two prevalent commercial brands (Dentsply Xive and Biomet 3i) as a proof-of-concept pipeline for mainstream systems. We acknowledge that identifying only two brands functionally represents a binary classification task. When encountering unfamiliar brands, the model may produce misclassifications. Consequently, future work will continuously expand the dataset by incorporating minority and less common implant systems, as well as multi-center cohorts with strict patient-level partitioning, to broaden the model’s diagnostic applicability and clinical knowledge base. Second, the inherent limitations of two-dimensional imaging should be considered. Periapical radiographs compress three-dimensional oral structures into a two-dimensional plane, resulting in overlapping structures or morphological distortions due to variations in projection angles. These factors, such as specific thread designs, may obscure the subtle features required for accurate implant brand recognition. To address this issue, future work will incorporate three-dimensional computed tomography (CT) imaging as an auxiliary diagnostic tool to enhance feature interpretation and classification accuracy. Third, the independent test set comprised a relatively small sample size (*n* = 61 implant instances across 51 radiographs), which introduces potential sensitivity where single-instance classification errors notably alter point estimates. While the 5-fold cross-validation mitigates split-dependent bias, future validations on expanded cohorts will corroborate these findings with higher statistical power. Finally, the goal of this research lies in clinical translation. We plan to develop the proposed model into a user-friendly software system, integrating visualization tools to present the rationale behind the model’s decisions to clinicians. In particular, implementing saliency-based explainability methods such as Grad-CAM will provide visual validation that predictions consistently rely on constructive implant features (such as platform connection margins and thread pitches) rather than background bone artifacts, thereby enhancing diagnostic transparency and facilitating chairside adoption in daily dental practice.

Additionally, the clinical comparison was conducted on a curated case series (*n* = 8 PAs), which primarily serves as a preliminary proof-of-concept for real-time inference feasibility; broader prospective clinical validations across diverse clinical setups will be pursued in future work. Specifically, a prospective multi-reader study on an independent clinical cohort—directly comparing diagnostic accuracy and reading duration with versus without AI assistance—is planned to evaluate true chairside utility.

## 5. Conclusions

This study developed an automated artificial intelligence-assisted diagnostic framework integrating YOLOv8 and EfficientNet-B3 to simultaneously identify dental implant brands and their corresponding exposure statuses using periapical radiographs. Addressing the clinical challenge of incomplete medical records, the two-step system demonstrated reliable diagnostic performance across six distinct categories. In the detection phase, YOLOv8 achieved 99.39% precision and 98.63% sensitivity. In subsequent classification, EfficientNet-B3 achieved an accuracy of 99.18 ± 0.85% (95% CI: [94.20%, 100.00%]) across 5-fold cross-validation. Crucially, preliminary evaluations indicated promising assistive diagnostic potential alongside participating clinicians, demonstrating rapid algorithmic execution with a mean inference time of 36.18 milliseconds per evaluation. Ultimately, this framework provides a viable proof-of-concept supported by robust internal validation; however, rigorous prospective clinical evaluations and multicenter external studies across expanded implant systems remain essential prior to routine clinical implementation.

## Figures and Tables

**Table 1:** Implant detection performance comparison across different YOLO architectures evaluated on the independent test set (*n* = 61 instances from 51 radiographs), alongside 5-fold cross-validation precision intervals.

Model	YOLOv8	YOLOv10	YOLOv11	YOLOv12
Precision	99.39%	99.13%	99.12%	99.02%
Sensitivity	98.63%	96.71%	98.62%	97.50%
F1-Score	98.88%	98.03%	98.87%	98.25%
mAP50	99.13%	98.94%	99.02%	99.07%
mAP50-95	80.05%	80.66%	81.10%	77.48%
Training Loss	0.52002	1.20741	0.58847	0.83976
Precision (95% CI)	[96.72%, 99.92%]	[95.95%, 99.88%]	[95.90%, 99.87%]	[95.70%, 99.84%]

Note: mAP50: mean Average Precision at an IoU threshold of 0.50; mAP50-95: mean Average Precision calculated across IoU thresholds from 0.50 to 0.95; CI: Confidence Interval. Point estimates (Precision, Sensitivity, and mAP) reflect optimal model checkpoints evaluated on the independent held-out test set, whereas 95% Confidence Intervals (CI) were derived from five-fold cross-validation across the complete dataset. Training loss values reflect internal convergence trajectories under each architecture’s native loss formulation and are not intended for direct cross-model absolute numerical comparison.

**Table 2:** Performance comparison with different classification models (5-fold cross-validation).

Model	Accuracy	Precision	Sensitivity	F1-Score	95% CI (Accuracy)
ResNet50	98.87 ± 0.95%	98.88 ± 0.92%	98.95 ± 0.88%	98.91 ± 0.90%	[93.50%, 99.80%]
DenseNet121	98.23 ± 1.15%	98.91 ± 0.85%	98.83 ± 0.90%	98.87 ± 0.87%	[92.40%, 99.70%]
MobileNet_v3	98.78 ± 1.02%	99.12 ± 0.78%	98.72 ± 0.95%	98.92 ± 0.86%	[93.30%, 99.80%]
EfficientNet-B3	99.18 ± 0.85%	99.25 ± 0.76%	99.10 ± 0.92%	99.17 ± 0.81%	[94.20%, 100.00%]
ConvNeXt_v2	98.36 ± 1.10%	98.33 ± 1.05%	97.22 ± 1.20%	97.77 ± 1.12%	[92.60%, 99.70%]

**Table 3:** Comparison of ground truth, dentists’ annotation, and AI inference on representative test cases (*n* = 8 PAs).

PA	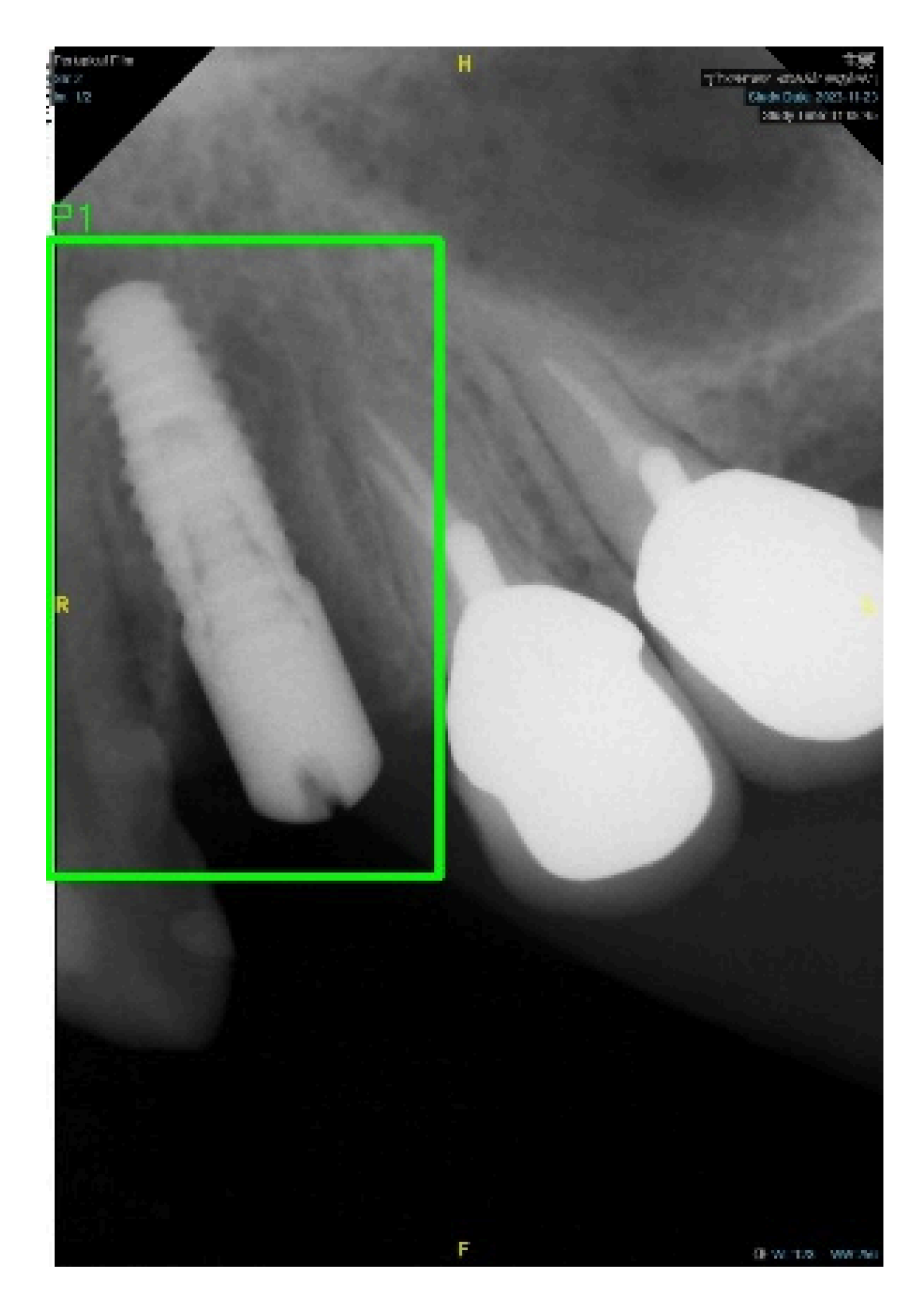	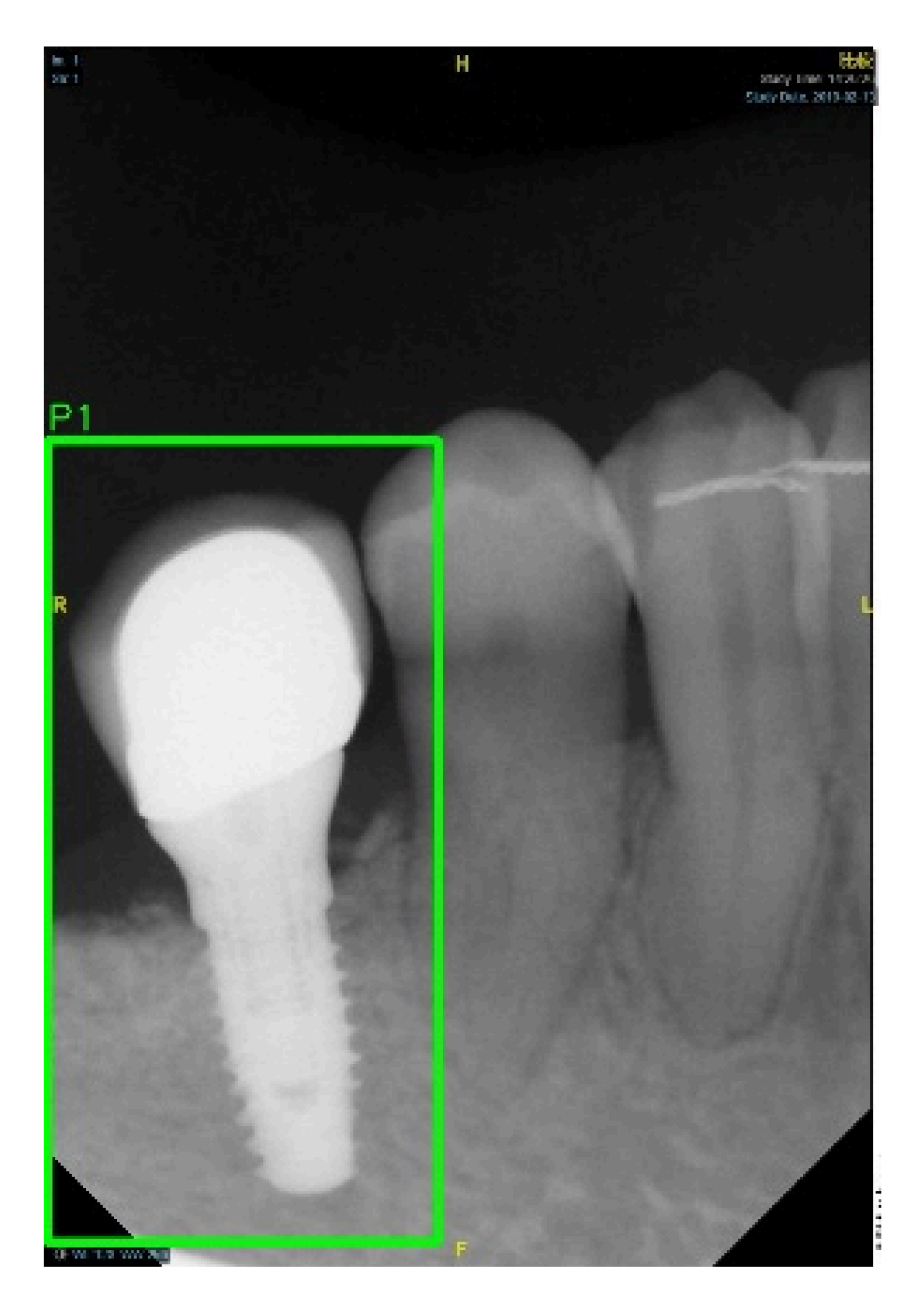	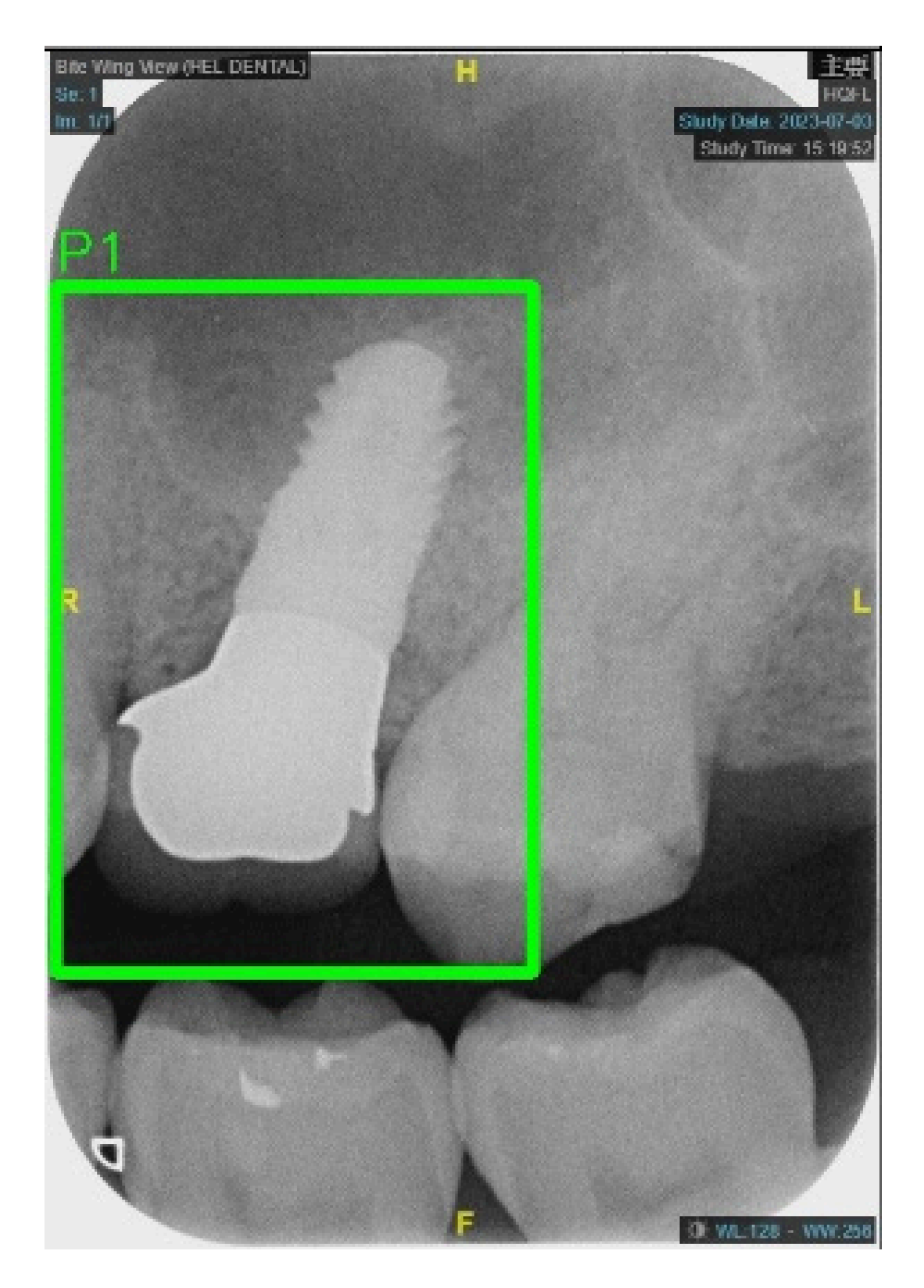	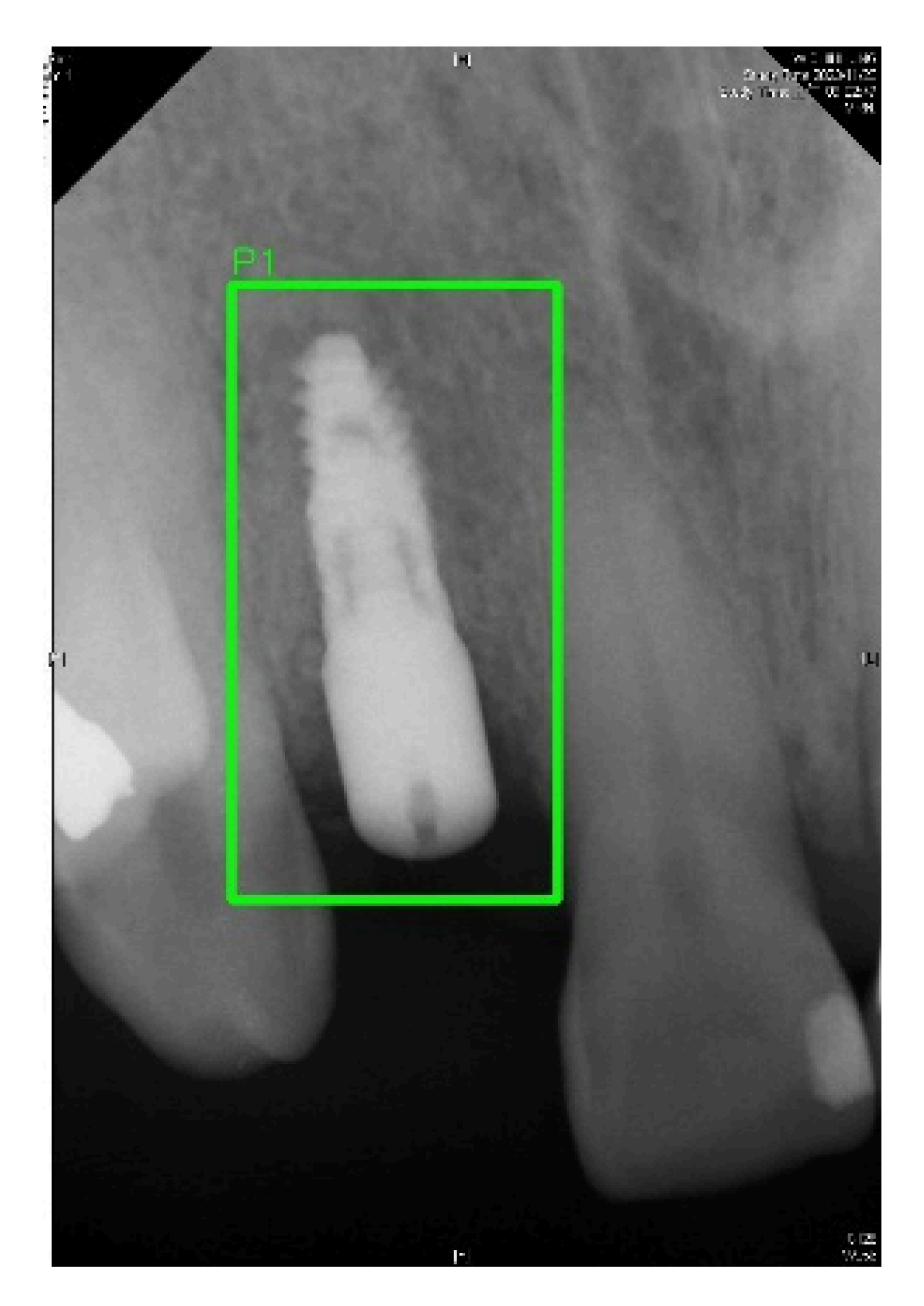
Ground truth (Implant brand/Status)	Xive/Healing Cap	3i/Prosthesis	Xive/Prosthesis	Xive/Healing Cap
Dentists classify results with confidence and required time on single implant				
Implant brand/Status	Xive/Healing Cap	3i/Prosthesis	Xive/Prosthesis	Xive/Healing Cap
Required time	12.94 s	15.93 s	5.69 s	13.04 s
Confidence	80%	85%	55%	70%
AI-assisted framework results and required time on multiple implants				
Implant brand/Status	Xive/Healing Cap	3i/Prosthesis	Xive/Prosthesis	Xive/Healing Cap
Inference Time	37.60 ms	31.86 ms	30.05 ms	35.43 ms
Mean Confidence Score	99.55%	99.84%	99.13%	99.87%
PA	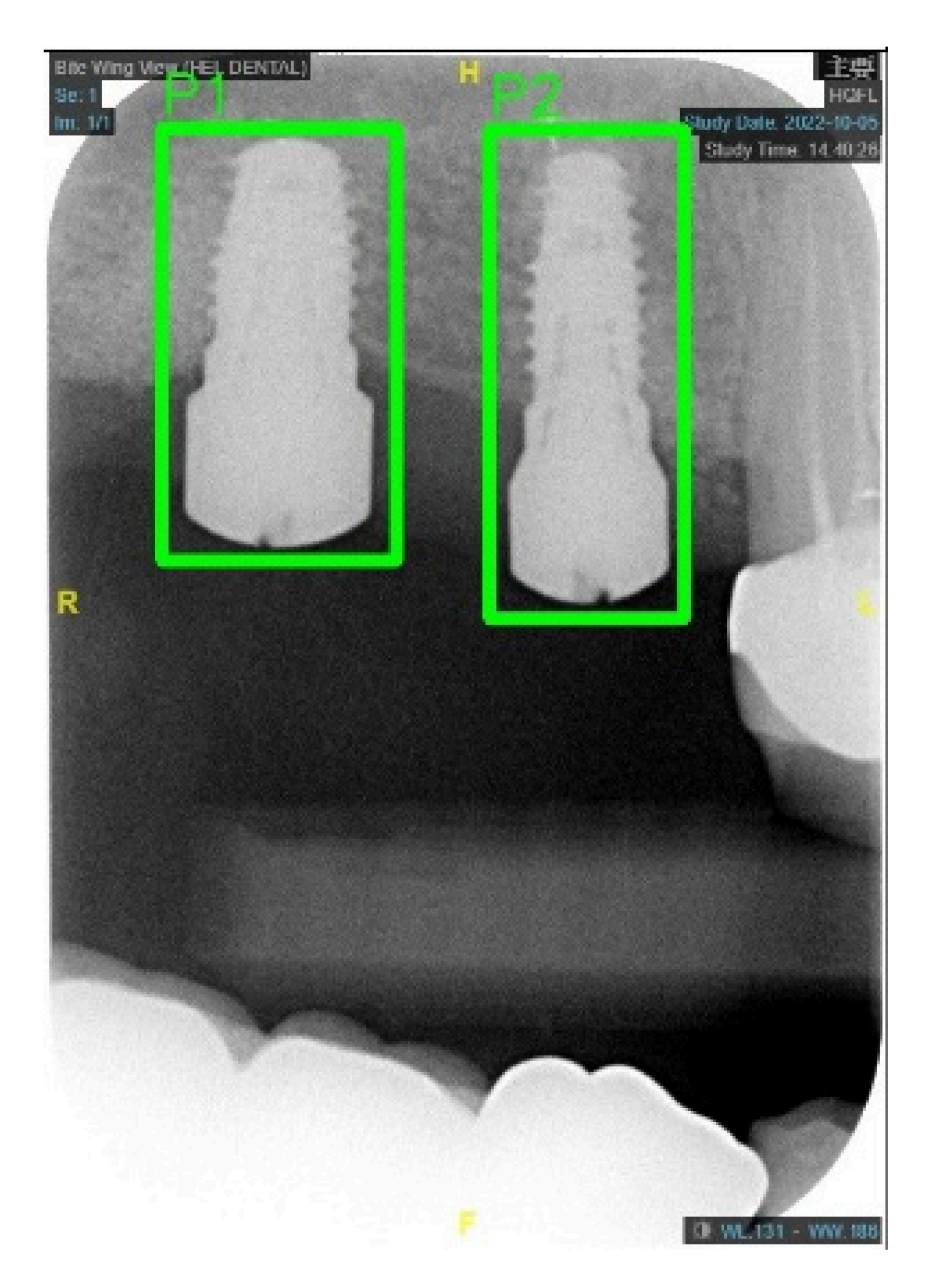	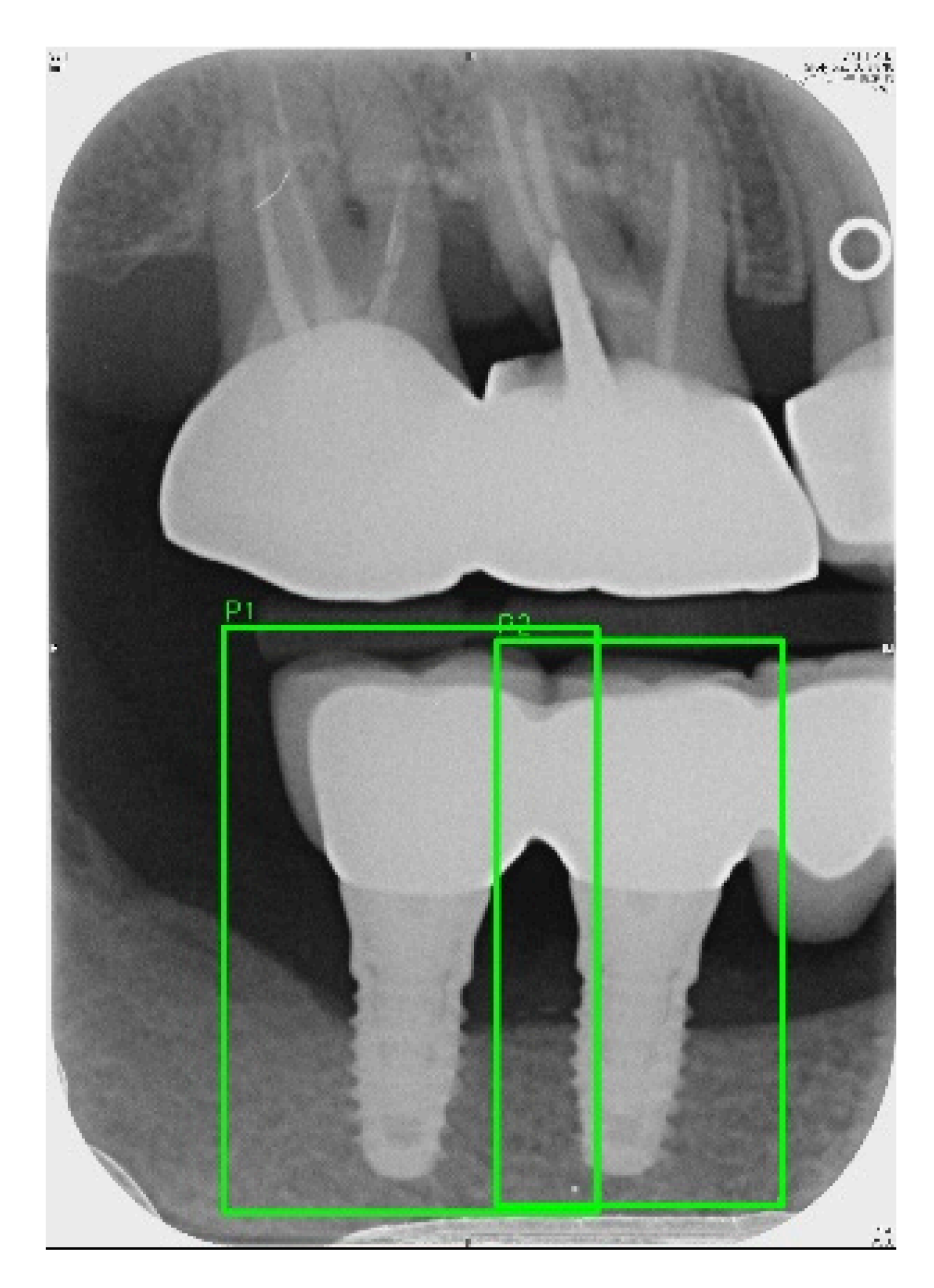	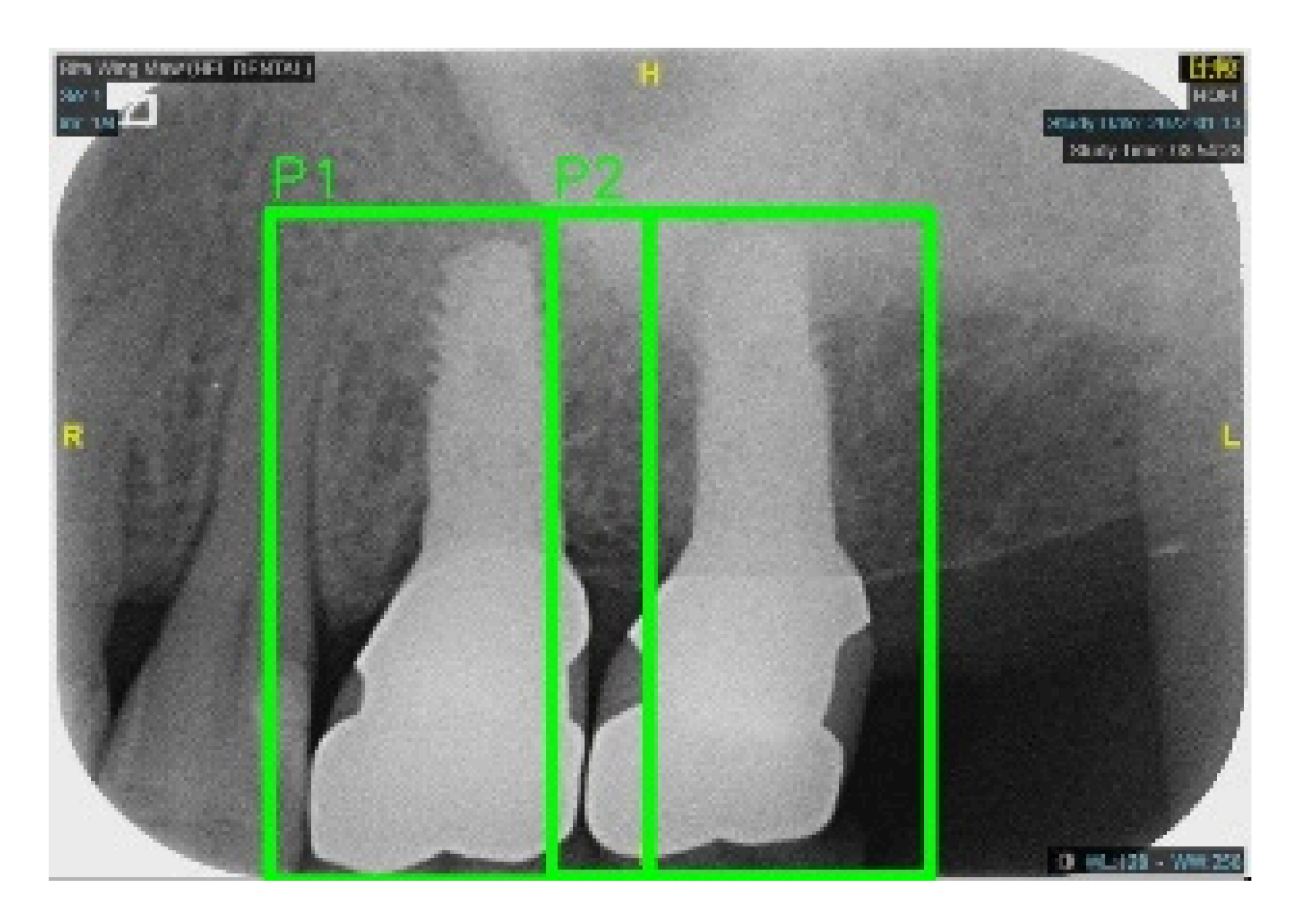	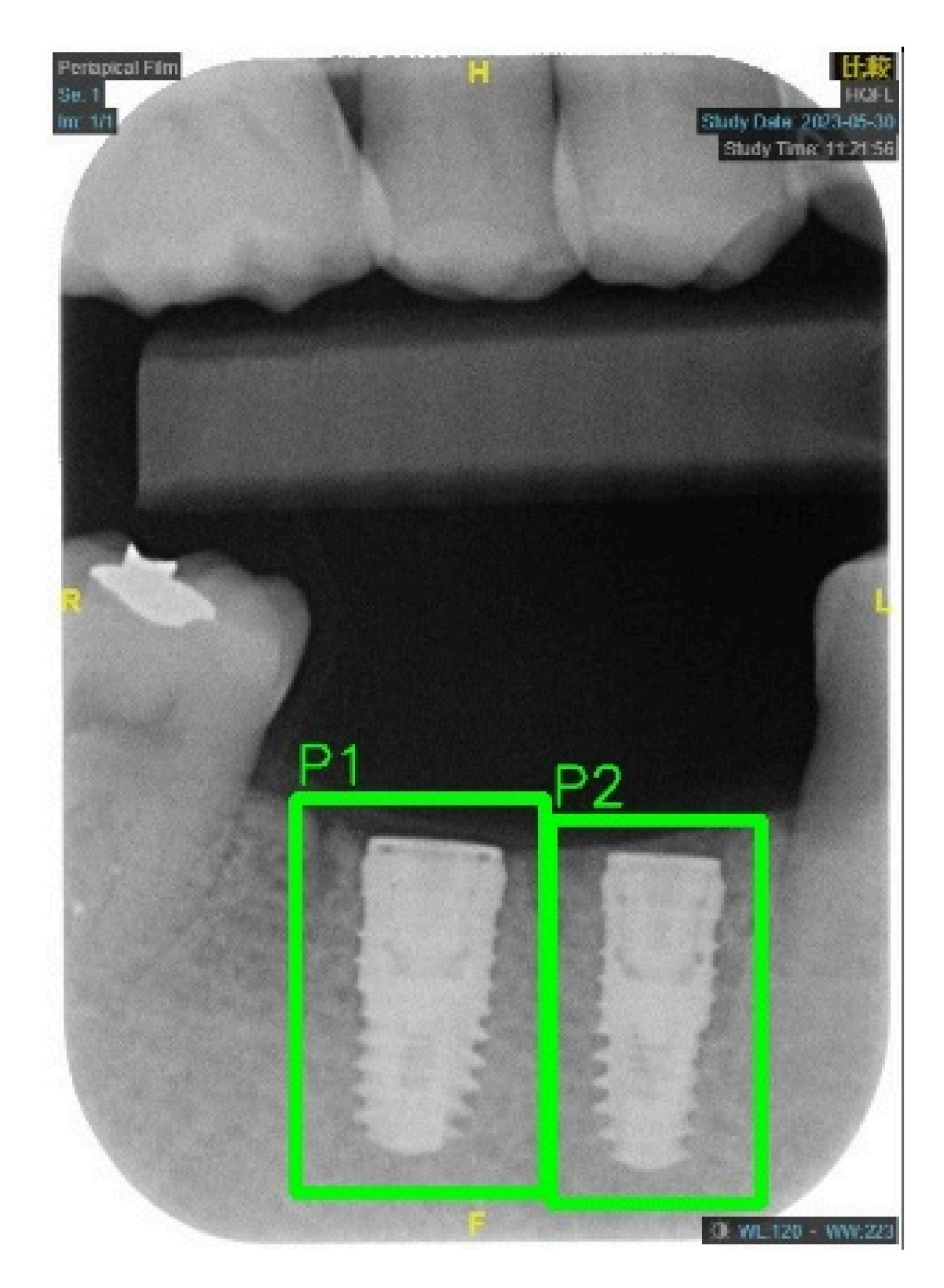
Ground truth (Implant brand/Status)	3i/Healing Cap	3i/Prosthesis	3i/Prosthesis	Xive/Emerged
Dentists classify results with confidence and required time on single implants				
Implant brand/Status	3i/Healing Cap	3i/Prosthesis	3i/Prosthesis	Xive/Emerged
Required time	5.93 s	5.14 s	6.04 s	5.51 s
Confidence	90%	70%	85%	85%
AI-assisted framework results and required time on multiple implants				
Implant brand/Status	3i/Healing Cap	3i/Prosthesis	3i/Prosthesis	Xive/Emerged
Inference Time	46.33 ms	49.07 ms	29.04 ms	30.05 ms
Mean Confidence Score	99.94%	99.87%	99.70%	99.88%

## Data Availability

The dataset can be made available from the corresponding authors upon reasonable request only for research purposes, subject to approval by the Institutional Review Board.
